# Uterine Injury Caused by Genotype 4 Hepatitis E Virus Infection Based on a BALB/c Mice Model

**DOI:** 10.3390/v13101950

**Published:** 2021-09-28

**Authors:** Weimin Yang, Shuangfeng Chen, Houfack K. Mickael, Liangheng Xu, Yueping Xia, Chao Cong, Yike Zhang, Zhongyao Qian, Tengyuan Li, Daqiao Wei, Wenhai Yu, Fen Huang

**Affiliations:** 1 Medical Faculty, Kunming University of Science and Technology, Kunming 650500, China; yangweimin7777@163.com (W.Y.); csf521csy6688@163.com (S.C.); houfack@gmail.com (H.K.M.); heng1178@163.com (L.X.); xyp68995@163.com (Y.X.); cc18469171503@163.com (C.C.); zhangyike77332@163.com (Y.Z.); qianzhongyao96@163.com (Z.Q.); ltylitengyuan@163.com (T.L.); weidaqiao1234@kmust.edu.cn (D.W.); 2 Institute of Medical Biology, Chinese Academy of Medical Sciences and Peking Union Medical College, Kunming 650038, China; wenhaiyu1234@163.com; 3 Yunnan Provincial Key Laboratory of Clinical Virology, Kunming 650034, China

**Keywords:** hepatitis E virus, pregnancy, uterine injury, miscarriage

## Abstract

To evaluate whether uterine injury caused by hepatitis E virus (HEV) infection is responsible for adverse pregnancy outcomes. HEV-infected female BALB/c mice were coupled with healthy male BALB/c mice at 0, 7, 14, 21, and 91 dpi to explore the uterine injury caused by HEV infection. Mice were euthanized after 10 days of copulation, and uteruses were collected for HEV RNA and antigen detection and histopathological analysis. Inflammatory responses; apoptosis; and estrogen receptor ɑ (ER-ɑ), endomethal antibody (ERAb), cytokeratin-7 (CK7), vimentin (VIM), and vascular endothelial growth factor (VEGF) expression levels were evaluated. After 10 days of copulation, miscarriage and nonpregnancy, as well as enlarged uteruses filled with inflammatory cytokines, were found in HEV-infected mice. HEV RNA and antigens were detected in the sera and uteruses of HEV-infected mice. Significant endometrial thickness (EMT) thinning, severe inflammatory responses, and aggravated apoptosis in the uteruses of HEV-infected mice that experienced miscarriage might contribute to adverse pregnancy outcomes. Furthermore, significantly suppressed ER-ɑ expression and increased ERAb, CK7, VIM, and VEGF expression levels were found in the uteruses of HEV-infected mice that had miscarried. However, uterine damage recovered after complete HEV clearance, and impaired fertility was improved. EMT injury, severe inflammatory responses, and aggravated apoptosis in the uterus caused by HEV infection are responsible for poor pregnancy outcomes.

## 1. Introduction

Hepatitis E virus (HEV) is one of the most common causes of acute hepatitis worldwide. It results in 20 million infections and 70,000 related deaths every year [1,2,3]. HEV genotype 1 and 2 infections cause large outbreaks and affect young subjects with a significant mortality rate in pregnant women and patients with cirrhosis in developing countries [4], while genotype 3 and 4 infections are responsible for sporadic and zoonotic diseases and are mainly transmitted by consumption of undercooked meat or milk in developed countries [5,6,7]. HEV infection is usually self-limiting and has a mortality of <1% in the general population [8]. However, genotype 1 HEV infection during pregnancy often results in fulminant hepatic failure (FHF) and leads to a maternal death rate of >25% [9]. Severe adverse pregnancy outcomes, including maternal death, premature delivery, abortion, and stillbirth, induced by HEV have been reported in the clinic [10,11]. Genotype 3 HEV infections are known to be less pathogenic at the maternal–fetal interface [12]. However, severe symptoms were induced by genotype 4 HEV infections [13]. Stillbirth, threatened preterm births, and abortion caused by HEV infection have been reported recently in China, where genotype 4 HEV is prevalent [14,15].

However, the pathogenesis of HEV infection during pregnancy is unclear. Extremely elevated estrogen and progesterone levels have been found in HEV-infected pregnant women [16,17]. The increase in hormones, especially estrogen, during pregnancy facilitates HEV replication through inhibition of the PI3K–AKT–mTOR and cAMPK–PKA–CREB signaling pathways [17,18]. Furthermore, the suppression of estrogen and progesterone receptors are associated with HEV replication during pregnancy [16,18,19]. In addition, the innate immunity of pregnant women shows increased sensitivity to HEV infection as reflected by elevated viral titers and prolonged viral infection duration [20]. Retinoic acid induces gene protein I (RIG-I), the most critical antiviral interferon-stimulated gene, is severely frustrated and is inhibited by 7460.70 ± 3421.28-fold in HEV-infected pregnant rhesus macaques relative to in nonpregnant rhesus macaques; this condition may be responsible for the impaired innate immune response against viral infection [20]. Disordered hormones, inhibited signal pathways, and frustrated innate immune systems may account for poor pregnancy outcomes.

Vertical transmission from mothers to fetuses is recognized as one of the routes of HEV transmission [20,21,22]. The uterus is the most important organ for the maintenance of pregnancy. The uterus, especially the endometrium lining, plays an important role in normal reproductive cycles, implantation, and placentation and in supporting a healthy fetus until parturition [23]. Pathogen infection in the uterus usually causes infertility, abortion, preterm delivery, and other clinical diseases [24,25,26,27]. The infection of the uterus by viruses, such as porcine reproductive and respiratory syndrome (PRRSV) [28], coxsackievirus B3 (Cvb3) [29], human papillomavirus [30], human cytomegalovirus [31,32], and Zika virus [33], results in adverse pregnancy outcomes. PRRSV replicates in the endometrium of sows and causes endometrial cell apoptosis, consequently leading to miscarriage, stillbirth, premature birth, neonatal death, and fetal hypoplasia [28]. Cvb3 infects the mouse uterus, produces an inflammatory response, and consequently results in spontaneous abortion [29]. Although genotype 4 HEV replication in the uteruses of HEV-infected BALB/c mice has been reported [34], the pathogenic mechanism of HEV infection in the uterus remains undefined.

In this work, genotype 4 HEV-infected BALB/c mice were used to mimic HEV infection during pregnancy to evaluate the pathogenesis of HEV infection in the uterus. Interestingly, failed pregnancy was found in most of the genotype 4 HEV-infected female mice even when they were synchronized with serum gonadotrophinum (PMSG) and chorionic gonadotrophin (HCG). Metritis, intimitis, and endometrium thinning were observed in the genotype 4 HEV-infected nonpregnant mice. However, the damages to the uterus recovered after HEV clearance, and impaired fertility partially improved.

## 2. Methods

### 2.1. Animal and Ethics Statement

BALB/c mice (female, 6 weeks old, *n* = 40) were purchased from Kunming Medical University and bred in a specific-pathogen-free facility at Kunming University of Science and Technology. All operations were performed in accordance with the guidelines of the Kunming University of Science and Technology Animal Care and Use Committee. Mice that were negative for anti-HEV antibodies (IgG and IgM) and HEV RNA were included in this study.

### 2.2. Reagents

PMSG (050074564) and HCG (050071281) were purchased from Chifeng Boen Pharmaceutical Co., Ltd. (Inner Mongolia, China). HEV-specific antibody (MAB8003) was obtained from Millipore (Burlington, MA, USA). Rabbit-anti-CD45+ (A2115) and ERAb (ab170864) were procured from ABclonal (Boston, MA, USA). Vimentin (Vim, D21H3) XP(R) was purchased from Cell Signaling Technology (Boston, MA, USA). Anti-F4/80+ antibody (ab6640), anticytokeratin 7 (ab181598), anti-VEGFA (ab1316), antiestrogen receptor alpha antibody (ab108398), and horseradish peroxidase- or fluorescein (FITC)-labeled secondary antibodies were purchased from Abcam (Cambridge, UK).

### 2.3. Virus

HEV genotype 4 (KM01, GenBank No. KJ155502) was collected from a village in Kunming, China. Fecal suspension (10%) was centrifuged at 12,000× *g* and 4 °C for 10 min, filtered through 0.22 µm microfilters, and treated with penicillin and streptomycin for 1 h. The suspension was then stored in liquid nitrogen until use. Viral genomic titers consisting of 1.0 × 10^6^ copies were determined by using quantitative real-time PCR (qRT-PCR) as previously described [35].

### 2.4. Cell Cultures and Viral Inoculation

Hela cells (human cervical cancer cell line) were maintained in Dulbecco’s modified Eagle’s medium (DMEM) supplemented with 10% fetal bovine serum at 37 ℃ with 5% CO_2_. The Hela cells were inoculated with HEV in accordance with our previous report [16]. The cells were harvested for HEV detection through qRT-PCR and immunofluorescence at indicated time points.

### 2.5. Experimental Design

**Figure 1:**
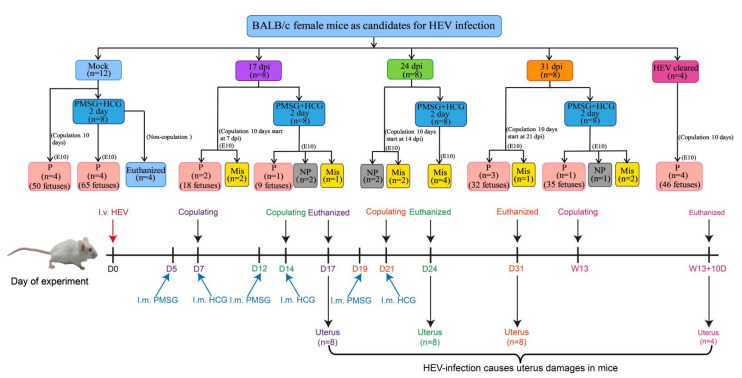
Diagram of HEV infection in female mice. P, pregnant; NP, nonpregnant; Mis, miscarried.

Mice were divided into five groups as shown in Figure 1. The mock group (*n* = 12) consisted of 12 mice. In the mock group, four mice that were coupled with healthy adult male mice served as the pregnancy control; four mice that were synchronized with PMSG and HCG by intramuscular injection after 24 h served as the synchronization group; and the remaining four mice that were injected with PBS without copulation served as the mock nonpregnant control. Mice that were intravenously infected with HEV at 7 days post-infection (dpi, *n* = 8), 14 dpi (*n* = 8), and 21 dpi (*n* = 8) were copulated with healthy adult male mice (1:1) for 10 days or pretreated with PMSG and HCG before 10 days of copulation (E10). Mice in the HEV-cleared group (*n* = 4, 91 dpi), which had been completely cleared of HEV RNA and antigens for at least for 56 days, were copulated with healthy adult male mice (1:1) to assess the effect of HEV infection after viral clearance. Serum samples were collected at 5, 8, and 10 days post-copulation for progesterone determination to identify whether the copulated mouse was pregnant. All mice were humanely euthanized and necropsied after 10 days of copulation (E10). Sera, spleens, and uteruses were collected, and the uteruses were fixed in neutral 4% paraformaldehyde solution.

### 2.6. HEV RNA Detection and Gene Quantification

Total RNA was extracted from sera, uteruses, or cells by Trizol (Invitrogen, CA, USA) in accordance with the manufacturer’s instructions. cDNA was prepared using AMV Reverse Transcriptase XL (Takara, Ibaraki, Japan) as the direction. Reverse transcription PCR was performed at 30 °C for 10 min, 42 °C for 30 min, 99 °C for 5 min, and 5 °C for 5 min. The genomic copy number of HEV was quantified using SYBR green-based qRT-PCR with HEV-specific primers as described in a previous study [5]. qRT-PCR was performed in the Bio-Rad CFX96TM Real-Time PCR System. GAPDH served as a loading control.

### 2.7. Morphological and Histopathological Analyses

Tissues for histopathological examination were fixed in 10% neutral buffered formalin, routinely processed, sectioned to a thickness of 3 µm, and then stained with hematoxylin and eosin (H&E). The samples were photographed and analyzed by using a microscope (Nikon, Tokyo, Japan) equipped with a digital camera.

### 2.8. Immunohistochemistry and Immunofluorescence

The tissue sections for immunohistochemical analysis (IHC) and immunofluorescence assay (IFA) were treated with 3% H_2_O_2_ for 10 min and blocked with 5% skimmed milk for 30 min. The sections were incubated with the indicated antibody at 4 °C overnight. After three cycles of washing with PBS, the sections were incubated with the secondary antibody as described in our previous report [36]. The tissue sections were viewed by using fluorescence microscopy (Nikon Ti-E, Tokyo, Japan).

For the IFA of cell cultures, the cells were fixed for 15 min with 4% paraformaldehyde at 48 h postinfection, incubated for 1 h at 37 °C with an HEV-specific antibody (MAB8003, Millipore, America), then inoculated with FITC-conjugated secondary antibodies for 45 min at room temperature. The cells were stained with DAPI for 15 min and viewed under a fluorescence microscope (Nikon Ti-E, Tokyo, Japan).

### 2.9. Terminal Deoxynucleotidyl Transferase dUTP Nick end Labeling Assay

Apoptotic cells in the uterus were evaluated in situ by using a One Step terminal deoxynucleotidyl transferase-mediated dUTP nick-end labelling (TUNEL) assay kit (Beyotime, C1086). Briefly, the tissue sections were deparaffinized, rehydrated, and then digested with DNase and proteinase K (20 μg/mL) for 30 min at 37 °C. The slides were incubated with the TUNEL detection kit for 60 min at 37 °C. Nuclei were counterstained with DAPI. Then, the TUNEL specimens were observed under a fluorescence microscope (Nikon Ti-E, Tokyo, Japan).

### 2.10. Statistical Analysis

All experiments were performed at least three times. Statistical analysis was performed by using IBM SPSS Statistics 21 software. The density of positive signals/bands of IHC or Western Blot was measured by Image-Pro Plus 6.0 software. Student’s *t*-test was performed to analyze the significance of differences between two groups: * *p* < 0.05; ** *p* < 0.01; *** *p* < 0.001.

## 3. Results

### 3.1. HEV Replicates in the Uterus

**Figure 2:**
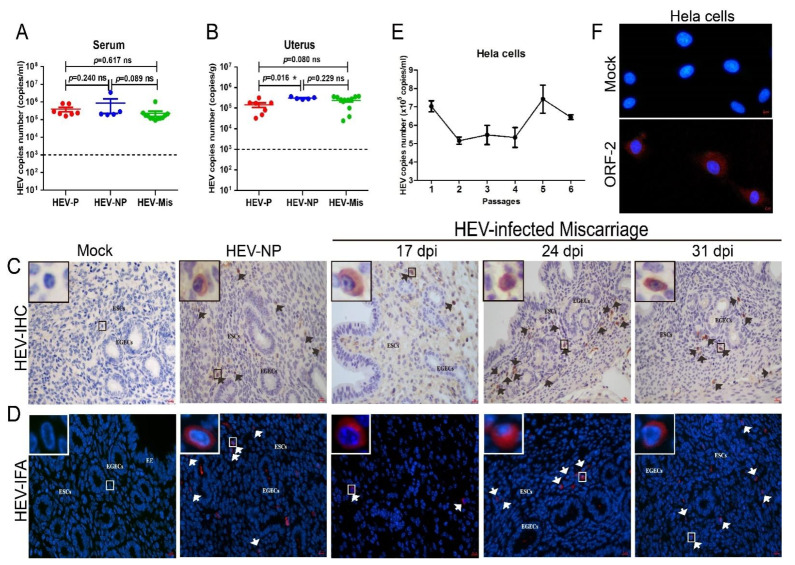
HEV replicates in the uterus. HEV RNA detected in the serum (**A**) and uterus (**B**) of HEV-infected mice and quantitated by qRT-PCR. The copy numbers of HEV RNA in the serum and uterus were compared and analyzed by using IBM SPSS Statistics 21 software. * *p* < 0.05. HEV antigens detected in the uteruses of mock or HEV-infected mice by IHC (**C**) and IFA (**D**). ESCs: endometrial stromal cells; EGECs: endometrial gland epithelial cells. (**E**) HEV titer in HEV-inoculated Hela cells determined by qRT-PCR. (**F**) HEV antigens (red) observed in mock or HEV-infected Hela cells by IFA with a HEV-specific antibody. Nuclei stained by DAPI (blue).

BALB/c mice with acute HEV infection were copulated with healthy male mice to evaluate the uterine injury caused by HEV infection and to investigate the pathogenesis of HEV infection in the uterus. The serum of all mice inoculated with HEV were positive for HEV RNA from 7 to 48 dpi (Figure 2A). Notably, HEV RNA was detected in the uteruses of HEV-infected mice with considerable serum viral titers (Figure 2B). This result indicated that HEV replicates in the uteruses of female mice. However, the sera and uteruses from mock mice were negative for HEV RNA. The uterus provides a safe space and adequate nutrition for fetal development and pregnancy maintenance [37]. IHC and IFA revealed the presence of HEV antigens in the uteruses of HEV-infected mice at 17, 24, and 31 dpi (Figure 2C,D). These results strongly demonstrated that the uterus is a replication site of HEV. Interestingly, HEV antigens mainly accumulated in the endometrium of infected mice.

Hela cells were inoculated with genotype 4 HEV to further identify the replication of HEV in the uterus. Notably, HEV RNA was persistently detectable in the cells for at least six passages (end of the experiment) with slowly increasing viral titer (Figure 2E). Meanwhile, the positive fluorescence signal of the HEV ORF2 capsid protein was observed in HEV-infected Hela cells at 48 h postinoculation, whereas no signal was found in uninfected mock cells (Figure 2F). These results strongly confirmed that HEV can replicate in the uterus.

### 3.2. Decreased Fertility in HEV-Infected Female Mice

**Figure 3:**
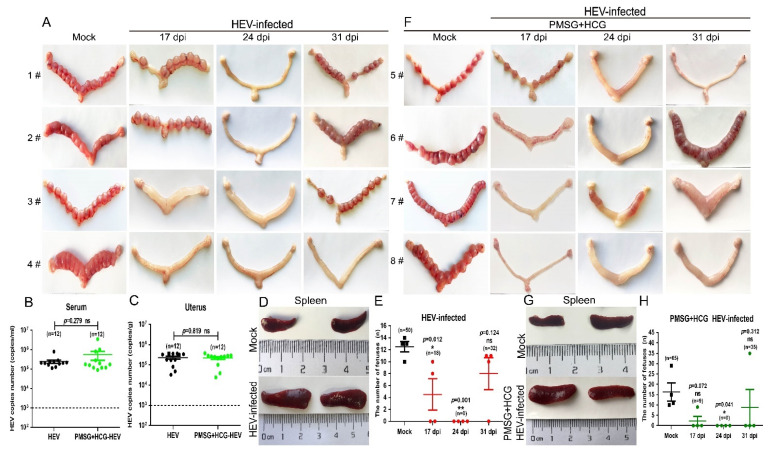
HEV infection results in decreased fertility in BALB/c female mice. (**A**) Morphological analysis of the uteruses from mock or HEV-infected mice at 17, 24, and 31 dpi (E 10). The copy numbers of HEV RNA in the serum (**B**) and uterus (**C**) of HEV-infected mice pretreated with or without PMSG and HCG were compared and analyzed by IBM SPSS Statistics 21 software. * *p* < 0.05; ** *p* < 0.01. (**D**) Sizes of spleens from mock or HEV-infected mothers. (**E**) Number of fetuses born to mock or HEV-infected mothers. (**F**) Morphological analysis of the uteruses from mock or HEV-infected mice synchronized with PMSG and HCG at 17, 24, and 31 dpi (E 10). (**G**) Sizes of spleens from mock or HEV-infected mothers who were synchronized with PMSG and HCG. (**H**) Number of fetuses born to mock or HEV-infected mothers who were synchronized with PMSG and HCG at 17, 24, and 31 dpi. Data were analyzed by IBM SPSS Statistics 21 software. * *p* < 0.05; ** *p* < 0.01.

HEV has been detected in infertile males with impaired sperm quality, and HEV infection causes pathological damage in the testis [36]. However, the HEV-induced impairment of reproduction in females is rarely evaluated. In the present study, each HEV-infected female mouse was separately copulated with at least three healthy adult male mice for 10 days. The body weight of female mice coupled with adult males were weighted every day after copulation (Supplementary Materials Figure S1), and the level of progesterone was determined at 5, 8, or 10 days post-copulation to decide whether the mouse was pregnant (Supplementary Materials Figure S2). After 10 days of copulation, all uninfected mock mice were found to be pregnant with a total of 48 fetuses. However, a significant reduction in pregnancy rates was found in HEV-infected mice at 17 (50% pregnant, 2/4), 24 (0 pregnant, 0/4), and 31 dpi (75% pregnant, 3/4) (Figure 3A). Moreover, the number of fetuses born to HEV-infected mothers also decreased; in particular, no fetuses were found in mice that were initially copulated at 14 dpi (Figure 3A,E).

Mice were pretreated with PMSG for 24 h and then injected with HCG to exclude the influence of estrum on pregnancy. The treatment of PMSG and HCG affected neither the viral replication in serum and uterus (Figure 3B,C) nor the splenomegaly (Figure 3D,G). However, the pregnancy rates of the HEV-infected mice did not improve (Figure 3F). Although more fetuses (fetuses = 65) were born to uninfected mock mice treated with PMSG and HCG than to mice in the untreated copulation control group (fetuses = 50), HEV-infected mice still bore only a few fetuses (9 fetuses in the 17 dpi group, 0 fetuses in the 24 dpi group, and 35 fetuses in the 31 dpi group) (Figure 3F,H). The significant decrement in the number of fetuses caused by HEV infection has also been reported in HEV-infected rabbits [38]. Thus, whether HEV infection impairs female reproduction must be urgently investigated in the clinic.

### 3.3. HEV Infection Results in Uterine Endometrial Damages and Evokes Inflammatory Responses in the Uteruses of Mice That Miscarried

**Figure 4:**
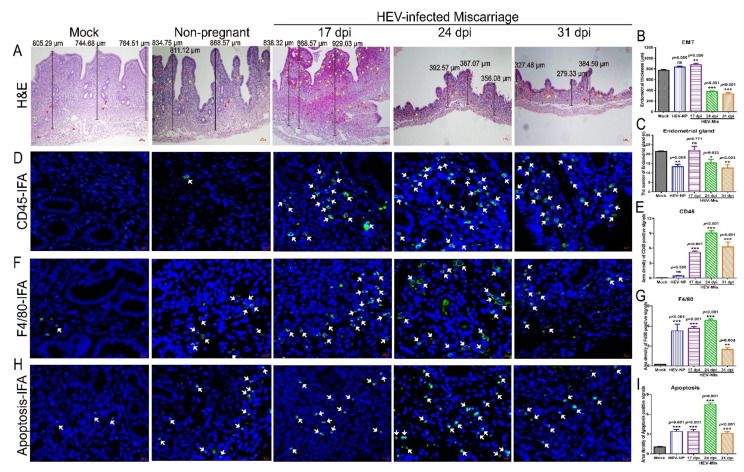
Uterine damages caused by HEV infection. (**A**) Histopathological analysis of uteruses from mock, HEV-infected nonpregnant, or HEV-infected miscarriage mice by H & E staining. (**B**) The EMT was measured and compared with that of mock mice, HEV-infected nonpregnant, or HEV-infected miscarriage mice at 17, 24, and 31 dpi. (**C**) The number of endometrial glands was calculated and compared with that of mock mice, HEV-infected nonpregnant, or HEV-infected miscarriage mice at 17, 24, and 31 dpi. The presence of CD45+ leukocytes ((**D**), white arrow), F4/80+ macrophages ((**F**), white arrow), and apoptotic cells ((**H**), white arrow) in the uteruses of mice with or without HEV infection were separately identified by IFA. Positive fluorescent signals of CD45+ leukocytes (**E**), F4/80+ macrophages (**G**), and apoptotic cells (**I**) were separately calculated by Image-Pro Plus 6.0 software and analyzed with IBM SPSS Statistics 21 software. * *p* < 0.05; ** *p* < 0.01; *** *p* < 0.001.

Infections with viruses, such as PRRSV and CVB3, have revealed that viral replication in the uterus is associated with tissue damage [22,23]. Endometrial thickness (EMT) is negatively related to the spontaneous abortion rate and ectopic pregnancy rate [39]. The EMT of HEV-infected mice that had experienced miscarriage was significantly thinner than that of the uninfected mock mice (Figure 4A,B) at 24 and 31 dpi. However, no significant difference was found in mice at 17 dpi. Meanwhile, loose myometrium, eosinophil infiltration, and significantly decrease endometrial glands were found in the HEV-infected miscarriage mice, especially in the mice in the 24 and 31 dpi groups (Figure 4A,C). Clinical diseases, such as metritis, endometritis, and abortion, are well-known important causes of infertility [23].

HEV infection usually inflicts damage via severe inflammatory responses. Inflammation is more severe in HEV-infected chimpanzees than in HCV-infected ones [40]. Thus, the inflammatory responses in the uterus were determined to evaluate the injury caused by HEV infection. Interestingly, significant increases in CD45+ leukocytes and F4/80+ macrophages were observed in the uteruses of HEV-infected mice at 24 or 31 dpi (Figure 4D–G). Meanwhile, a large number of fluorescent apoptotic signals were detected in the uteruses of HEV-infected mice, especially in mice infected with HEV for 24 days (Figure 4H,I). Elevated inflammatory responses and extensive apoptosis should be responsible for endometrial injury.

### 3.4. HEV Infection Causes Disordered Gene Expression in the Uteruses of Nonpregnant Mice

**Figure 5:**
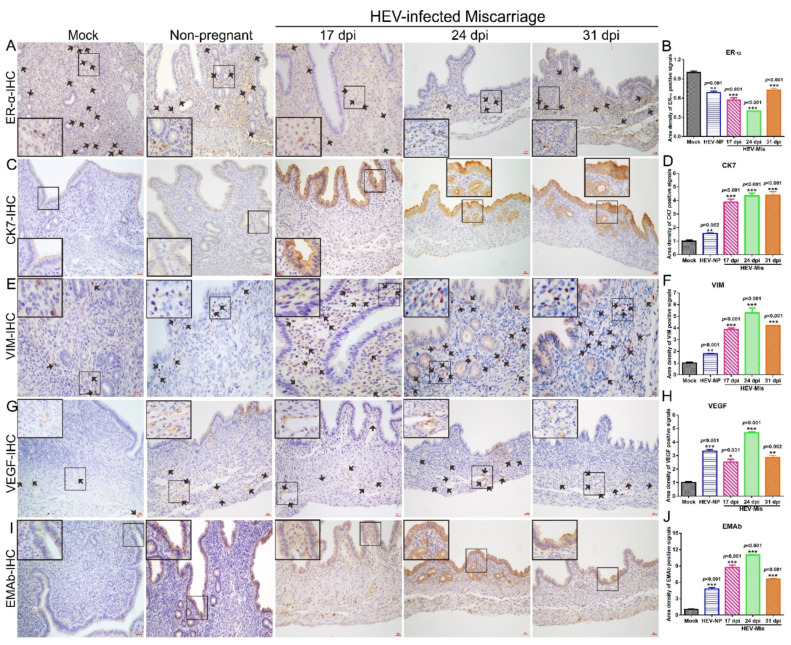
HEV infection causes gene disorders in mice that had miscarried. The expressions of ER-α (**A**), CK-7 (**C**), VIM (**E**), VEGF (**G**), and EMAb (**I**) in the uteruses of mock mice, HEV-infected nonpregnant mice, or HEV-infected mice that had miscarried at 17, 24, or 31 dpi were determined by IHC. Positive signals of ERα (**B**), CK7 (**D**), VIM (**F**), VEGF (**H**), and EMAb (**J**) in the uteruses of mock mice, HEV-infected nonpregnant mice, and HEV-infected miscarriage mice were separately calculated by Image-Pro Plus 6.0 software and compared by IBM SPSS Statistics 21 software. * *p* < 0.05; ** *p* < 0.01; *** *p* < 0.001.

**Figure 6:**
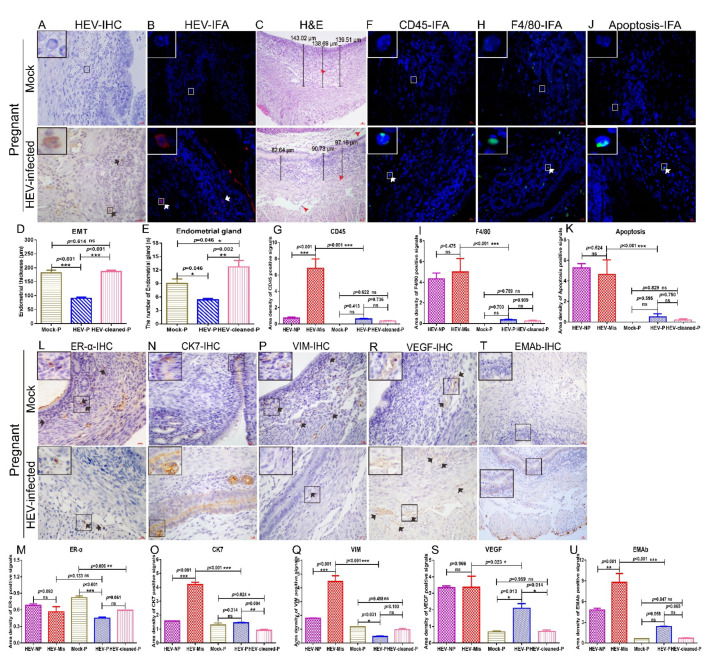
Uterine damages in HEV-infected pregnant mice are mild. HEV antigens were detected in the uteruses of mock or HEV-infected pregnant mice by IHC (**A**) and IFA (**B**). (**C**) H & E analysis of uteruses from mock pregnant mice or HEV-infected pregnant mice. The presence of CD45 (**F**), F4/80 (**H**), and apoptotic cells (**J**) in the uteruses of mock pregnant mice or HEV-infected pregnant mice were identified by IFA. Nuclei were stained with DAPI. (**D**) The EMT of HEV-infected mice was measured and compared with that of mock pregnant, HEV-infected pregnant mice, or mice that became pregnant after HEV clearance. (**E**) The number of endometrial glands was calculated and compared with that of mock pregnant mice, HEV-infected pregnant mice, or mice that became pregnant after HEV clearance (**E**). Positive signals of CD45 (**G**), F4/80 (**I**), and apoptotic cells (**K**) in the uteruses of mock pregnant mice, HEV-infected pregnant mice, and mice that became pregnant after HEV clearance were separately calculated by Image-Pro Plus 6.0 software and compared by IBM SPSS Statistics 21 software. * *p* < 0.05; ** *p* < 0.01; *** *p* < 0.001. The expressions of ERα (**L**), CK7 (**N**), VIM (**P**), VEGF (**R**), and EMAb (**T**) in the uteruses of mock pregnant mice or HEV-infected pregnant mice were determined by IHC. Positive signals of ERα (**M**), CK7 (**O**), VIM (**Q**), VEGF (**S**), and EMAb (**U**) in the uteruses of mock pregnant mice, HEV-infected pregnant or nonpregnant mice, and mice that became pregnant after HEV clearance were separately calculated by Image-Pro Plus 6.0 software and compared by IBM SPSS Statistics 21 software. * *p* < 0.05; ** *p* < 0.01; *** *p* < 0.001.

Estrogen plays an important role in maintaining pregnancy by binding to estrogen receptors. HEV has been confirmed to inhibit the expression of estrogen receptors (ER-α and ER-β) in HEV-infected cells [16]. In the present study, IHC analysis revealed that ER-α was significantly suppressed in the uteruses of all HEV-infected mice, regardless of whether they were miscarried, nonpregnant (Figure 5A,B), or nonpregnant mice (Figure 6L,M). These results strongly suggested that HEV infection reduces ER expression.

The endometrium is one of the essential components of the uterus [41]. The physiological functions of the uterine endometrium (uterine lining) include preparation for implantation and pregnancy maintenance [42]. Cytokeratin-7 (CK7), an epithelial marker, is also an inflammatory indicator that transduces signals and participates in inflammation development [43]. The expression of CK7 is positively correlated with the pathological grade of cervical intraepithelial neoplasia [44,45]. Therefore, the expression level of CK7 in the uteruses of HEV-infected mice was determined by IHC. The results showed that CK7 expression in the uteruses of HEV-infected mice that had undergone miscarriage was significantly increased relative to that in uninfected mock mice (Figure 5C,D). Meanwhile, CK7 expression in HEV-infected mice that had experienced miscarriage was higher than that in HEV-infected pregnant mice or in mice after HEV clearance (Figure 6N,O).

VIM, the most important intermediate fiber in mesenchymal cells, forms a network of cell scaffolds with microtubules and microfilaments to maintain the integrity of mesenchymal cells [46]. In addition, it is associated with various autoimmune diseases and organ transplantation immunity and has a nonspecific immune activation function that can promote inflammatory responses [47,48]. Interestingly, the expression of VIM in the uteruses of HEV-infected mice that had experienced miscarriage persistently increased relative to that in the uteruses of uninfected mock mice (Figure 5E,F). Similar to CK7, VIM was significantly expressed in HEV-infected mice that had miscarried relative to in HEV-infected pregnant mice (Figure 6P,Q).

VEGF is a vital angiogenesis factor that promotes the formation of new blood vessels [49]. The drastic increase in VEGF expression in patients infected with *Helicobacter pylori* suggests that VEGF expression is associated with inflammatory responses and persistent gastric mucosa injury [50]. In the present study, at 17, 24, or 31 dpi, VEGF expression in HEV-infected mice that had miscarried was distinctly activated (Figure 5G,H) relative to that in uninfected mock mice or HEV-infected pregnant mice (Figure 6R,S).

Abnormal autoimmune processes can affect reproduction at various stages. EMAb is an autoantibody that targets the endometrium and causes a series of immune responses. It can specifically bind to antigens, activate the complementary system, affect the implantation of fertilized eggs, and cause miscarriage [51]. EMAb levels are associated with the outcome of early threatened abortion. Therefore, the expression of EMAb in the uteruses of HEV-infected mice that had miscarried and pregnant mice were determined via IHC. Remarkably, EMAb was prominently expressed in mice that had experienced miscarriage (Figure 5I,J) relative to in uninfected mock mice or HEV-infected pregnant mice (Figure 6T,U). Taken together, the remarkably increased expression levels of CK7, VIM, VEGF, and EMAb and the prominent suppression of ER-α in the uteruses of HEV-infected mice that had miscarried than in uninfected mock mice or HEV-infected pregnant mice may contribute to unsuccessful pregnancies.

### 3.5. HEV Infection Induces Moderate Uterine Damages in Pregnant Mice

The HEV titer in the blood and uterus were quantified to understand why some HEV-infected mice could become pregnant, whereas others became sterile. Notably, serum viral titers did not significantly differ between pregnant mice and nonpregnant mice (Figure 2A). However, the viral titer in the uteruses of HEV-infected nonpregnant mice was significantly higher than that in the uteruses of HEV-infected pregnant mice (Figure 2B). These results strongly suggested that high viral titer in the uterus may be responsible for sterility. Although IHC and IFA revealed the presence of HEV antigens in the uteruses of HEV-infected pregnant mice (Figure 6A,B), the positive signals in these mice were weaker than those in mice that had miscarried (Figure 2C,D). However, the EMT of HEV-infected pregnant mice was significantly thinner than that of uninfected pregnant mice (Figure 6C,D). Histopathological analyses indicated that the uteruses of HEV-infected pregnant mice were infiltrated with few eosinophilic inflammation factors (Figure 6C). The EMT of HEV-infected pregnant mice was still significantly thinner than that of the uninfected mock pregnant mice or pregnant mice after complete HEV clearance (Figure 6C,D and Supplementary Materials Figure S3E). However, the number of endometrial glands in the uterus in HEV-infected pregnant mice did not significantly decrease compared with that in uninfected mock pregnant mice or mice that became pregnant after HEV clearance (Figure 6C,E and Supplementary Materials Figure S3E). Although sporadic CD45+ leukocytes (Figure 6F,G), F4/80+ macrophages (Figure 6H,I), and apoptotic cells (Figure 6J,K) were observed in the uteruses of HEV-infected pregnant mice, the number of positive cells in these groups of mice were significantly lower than that in HEV-infected mice that had experienced miscarriages (Figure 6G,I,K). These results indicated that the severe inflammatory responses in the uterus evoked by HEV infection may contribute to miscarriage. Furthermore, consistent with the results of our previous studies [16,52], IHC analysis revealed reduced ER-α expression in the uteruses of HEV-infected mice (Figure 6L,M). However, the expression levels of CK7, VIM, VEGF, and EMAb in the uteruses of HEV-infected pregnant mice were significantly lower than those in the uteruses of HEV-infected mice that had miscarried and were comparable with those in the uteruses of uninfected mock pregnant mice or pregnant mice after HEV clearance (Figure 6N–U). Uterine damage in HEV-infected pregnant mice was milder than that in HEV-infected mice that had miscarried.

### 3.6. HEV-Induced Uterine Injury Can Be Eliminated

Male genital tract damage caused by HEV infection can be partly recovered when the virus has been completely cleared from the body [4]. Given that HEV infection usually causes an acute disease, the recovery of the uterus after viral clearance was investigated. Female mice (*n* = 8) were coupled with healthy male mice (*n* = 8) to evaluate uterine injury after viral clearance. The existence of HEV antigens was excluded by IHC and IFA (Supplementary Materials Figure S3C,D). Mock-infected female mice (*n* = 4) were mated with normal male mice (*n* = 4) as the control. Interestingly, the mock and HEV-cleared mice were pregnant with a similar number of fetuses (48 fetuses in mock group vs. 46 fetuses in the HEV-cleared group [Supplementary Materials Figure S3A,B]). Pathological analysis indicated that the uteruses of HEV-cleared female mice lacked significant injury and had comparable EMT with those of mock mice (Supplementary Materials Figures S3E and S6D). Meanwhile, inflammatory responses were investigated in HEV-cleared pregnant mice. Notably, CD45+ leukocytes, F4/80+ macrophages, and apoptotic cells were very rare in the uteruses of HEV-cleared pregnant mice (Supplementary Materials Figures S3F–H and S6G,I,Q). Moreover, the expression levels of ER-ɑ, CK7, VIM, VEGF, and EMAb in HEV-cleared pregnant mice were not significantly different from those in mock pregnant mice (Supplementary Materials Figures S3I–M and S6M,O,Q,S,U). These results indicated that the uterine damage caused by acute HEV infection can be recovered after complete HEV clearance.

## 4. Discussion

Although HEV infection usually produces acute and self-limiting diseases in the general population, it results in serious morbidity and mortality during pregnancy [53]. HEV infection during pregnancy causes adverse pregnancy outcomes, such as acute liver failure, FHF, hemorrhage, miscarriage, stillbirth, and preterm delivery [14]. Although HEV mainly replicates in the liver, multiple extrahepatic sites, such as the brain, spleen, intestines, kidney, muscles, and testes, have been reported [36,54,55,56]. In vitro studies using the decidua basalis and fetal placenta have found that HEV replicates in the human maternal–fetal interface [12]. The presence of HEV RNA and antigens in the placenta, uterus, and ovaries indicates that HEV can replicate in the female reproductive system [20,34,57,58]. However, the exact pathogenesis of HEV during pregnancy remains to be defined.

Approximately 8–16% of miscarriages occur in HEV-infected pregnant women [59,60]. Nevertheless, the pathogenesis of this condition is largely unknown. Pregnancy is a complex process for maintaining fetal development in the uterus. Any damage to the uterus caused by pathogen infection threatens the fetus [61]. BALB/c mice are sensitive to genotype 4 HEV but dull to genotype 3 HEV, which only infects humanized mice [62,63]. Concerns have been raised about the adverse pregnancy outcomes caused by genotype 4 HEV infection. In the present study, genotype 4 HEV-infected BALB/c mice were used to explore the uterus damages caused by HEV infection. Notably, miscarriage or infertility was found in 58.33% (7/12) of HEV-infected mice. Worsened pregnancy outcomes were obtained in HEV-infected mice treated with PMSG and HCG for estrus synchronization, and only one mouse was found to be pregnant (1/12). The serum concentration of progesterone first increased and then decreased strongly. This trend indicated that abortion was caused by HEV infection. The significant reduction in the number of fetuses at 24 dpi also suggested adverse pregnancy outcomes during HEV infection. The comparable viral titers in the uterus and serum implied that HEV efficiently replicates in the uterus. Pathological analysis revealed an extremely thin EMT filled with CD45^+^ leukocytes and F4/80^+^ macrophages. Apoptosis was significantly increased in the uteruses of HEV-infected nonpregnant mice from 17 to 30 dpi (end of the experiment). Efficient viral replication in the uterus may have contributed to severe inflammation and cell apoptosis and then resulted in EMT damage, which is harmful to the maintenance of pregnancy. Interestingly, although no significant differences were observed among the serum viral titers of HEV-infected nonpregnant mice, mice that had miscarried, and pregnant mice, the viral titers in the uteruses of nonpregnant mice were significantly higher than those in pregnant mice. Meanwhile, although the EMT in HEV-infected pregnant mice appeared normal compared with that in uninfected mock pregnant mice, the EMT of HEV-infected nonpregnant mice, mice that had miscarried, and pregnant mice were all significantly thinner than that of uninfected mock mice. The impairment of the EMT and endometrial gland strongly suggested that HEV infection results in severe EMT damage. However, the EMT and endometrial gland recovered after complete HEV clearance, and normal pregnancy was observed. The results suggested that the EMT damage caused by HEV is responsible for the failure of pregnancy maintenance. In addition, inflammatory responses and cell apoptosis were significantly more severe in the uteruses of HEV-infected mice that had experienced miscarriage than in the uteruses of pregnant mice with or without HEV. This result further indicated that the serious impairment of EMT contributes to poor pregnancy outcomes.

HEV infection in the uterus may decrease uterine vitality and cause pathological damage. Moreover, the expression levels of ERAb, CK7, VIM, and VEGF were significantly increased in the uteruses of HEV-infected mice that had miscarried compared with those in the uteruses of HEV-infected pregnant mice. This outcome indicated that HEV infection promoted the expression of ERAb, CK7, VIM, and VEGF and accelerated adverse pregnancy outcomes. The inhibition of ER-α expression by HEV infection has been confirmed in pregnant women and HEV cell cultures [16,17]. In the present study, ER-α expression was significantly suppressed in the uteruses of HEV-infected pregnant mice or mice that had experienced miscarriage. The reduction in ER-α expression inhibited the phosphorylation of S6 in the PI3K–AKT–mTOR signaling pathway and subsequently increased HEV replication [18]. Our previous studies have shown that HEV infection regulates estrogen signaling pathways by inhibiting the cAMPK–PKA–CREB and PI3K–AKT–mTOR signaling pathways [17,18]. Thus, the dysregulation of genes in the uterus may be responsible for adverse pregnancy outcomes. Notably, worsened pregnancy outcomes were observed in HEV-infected mice treated with PMSG and HCG for estrus synchronization. Excess hormones may accelerate HEV replication and result in endocrine disorders. Thus, couples who are undergoing in vitro fertilization should be screened for HEV infection.

## 5. Conclusions

HEV replicates in the uterus and results in abortion and infertility. Severe injuries with significant EMT thinning, increased inflammatory responses, and aggravated apoptosis were found in the uteruses of HEV-infected mice that had experienced miscarriage. The suppression of ER-α expression and the increase in CK7, ERAb, VIM, and VEGF expression caused by HEV infection may contribute to adverse pregnancy outcomes. These findings have important implications for our understanding of the pathogenesis of HEV during pregnancy.

## Data Availability

The data that support the findings of this study are available within the article as well as in the supplementary files.

## Supplementary Materials

The following are available online at https://www.mdpi.com/article/10.3390/v13101950/s1, Figure S1: The body weight changes in mock or HEV-infected female mice from mating for 10 days. Figure S2: The concentration of progesterone in mock or HEV infected female mice. Figure S3: Uterine environment of pregnant mice after HEV-clearance.
