# Supplementary material for: Uterine Injury Caused by Genotype 4 Hepatitis E Virus Infection Based on a BALB/c Mice Model
Source: Viruses. 2021 Sep 28;13(10):1950. doi: 10.3390/v13101950 (PMC8538062; doi:10.3390/v13101950)
Supplement: Supplementary file 1 [file viruses-13-01950-s001.zip › viruses-1376890-supplementary.pdf]

viruses

Supplementary date

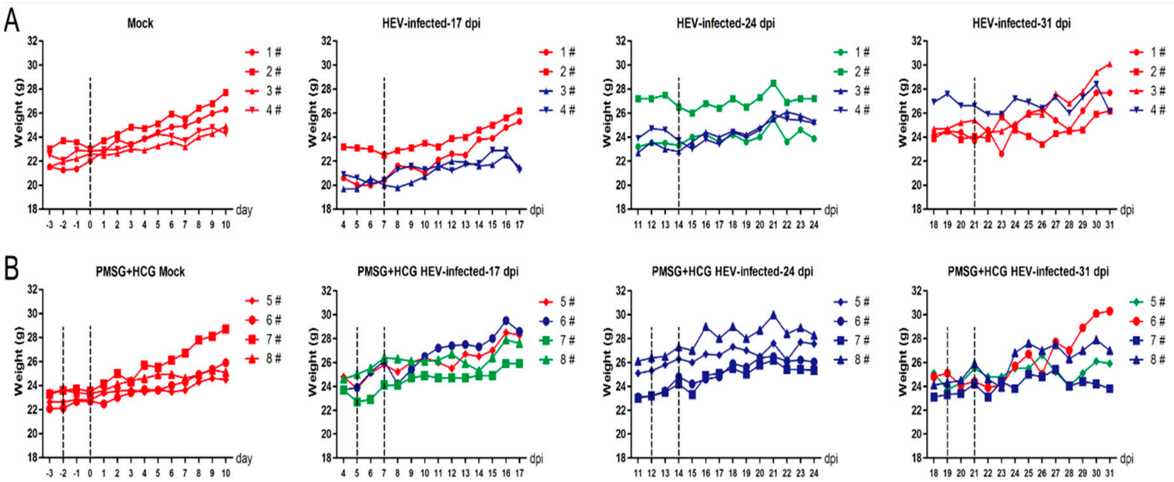

Figure S1. The body weight changes in mock or HEV-infected female mice from mating for 10 days. (**A**) Body weight of mock or HEV-infected female mice. The dotted lines indicated that the mice were copulated with normal male mice for 10 days at 7, 14 and 21 dpi, respectively. (**B**) Body weight of PMSG+HCG treatment mock or HEV infected female mice. The first dotted lines showed that PMSG treatment for female mice, and the second dashed line showed that the female mice were i.m. HCG at 7, 14 and 21 dpi, respectively, and then copulated with normal male mice for 10 days. Non-pregnant mice are represented by green, miscarriage mice are represented by blue and pregnancy miceare represented by red.

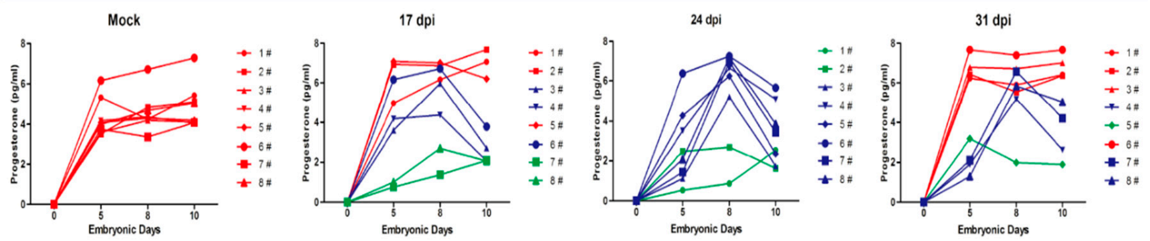

Figure S2. The concentration of progesterone in mock or HEV infected female mice. Non-pregnant mice are represented by green, miscarriage mice are represented by blue and pregnancy mice are represented by red.

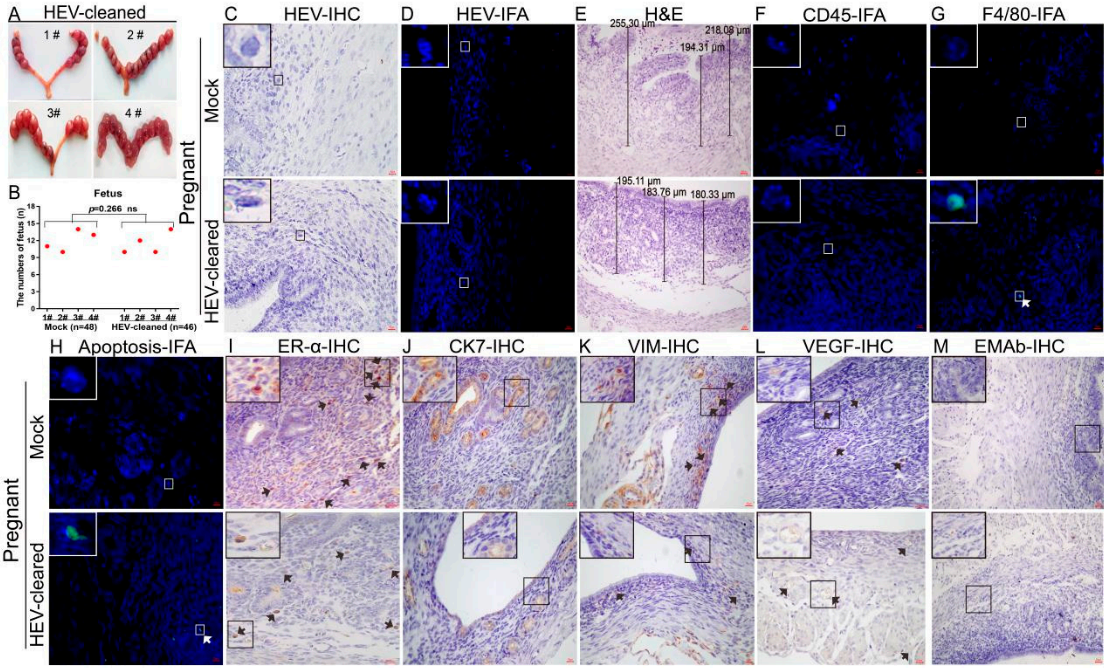

Figure S3. Uterine environment of pregnant mice after HEV-clearance. (**A**) Pregnancy outcomes of HEV-cleaned female mice (E10). (**B**) The number of fetus from HEV infected mother corresponds to the results of Supplementary Fig. 3 A. HEV antigens detected in the uterus of mock or HEV-infected pregnant mice by IHC (**C**) and IFA (**D**). (**E**) H&E analysis of uterus from mock or HEV-cleaned pregnant mice (Red arrowheads: eosinophilic granulocyte). (**F–H**) IFA of CD45+, F4/80+ and TUNEL apoptosis (green) in the uterus from mock or HEV-cleaned pregnant mice. (**I–M**) IHC of ER-α, CK7, VIM, VEGF and EMAb (arrow) in the uterus from mock or HEV-cleaned pregnant mice. Data were analyzed by IBM SPSS Statistics 21 software. *p<0.05; ** p<0.01; ***p<0.001.
